# Targeted Screening of Lactic Acid Bacteria With Antibacterial Activity Toward *Staphylococcus aureus* Clonal Complex Type 1 Associated With Atopic Dermatitis

**DOI:** 10.3389/fmicb.2021.733847

**Published:** 2021-09-17

**Authors:** Ida B. Christensen, Charlotte Vedel, Maja-Lisa Clausen, Søren Kjærulff, Tove Agner, Dennis S. Nielsen

**Affiliations:** ^1^Lactobio A/S, Copenhagen, Denmark; ^2^Department of Dermatology, Bispebjerg Hospital, University of Copenhagen, Copenhagen, Denmark; ^3^Department of Food Science, Faculty of Science, University of Copenhagen, Copenhagen, Denmark

**Keywords:** lactobacilli, antimicrobial compounds, inhibitory activity, skin, atopic dermatitis, *Staphylococcus aureus*

## Abstract

Atopic dermatitis (AD) is a common inflammatory skin disease characterized by an epidermal barrier impairment, as well as a Th2/Th22-skewed immune response, both favoring skin colonization with *Staphylococcus aureus*. Colonization is strongly related to severity of the disease, and a reduction of *S. aureus* has been found to alleviate symptoms. Lactic acid bacteria (LAB) produce antimicrobial compounds such as organic acids and bacteriocins and are widely used as probiotics. The aim of this study was to isolate LAB and screen for antibacterial effect specifically toward *S. aureus* clonal complex type 1. A total of 680 LAB were isolated from fermented vegetables and swab samples from healthy volunteers (vaginal, stool and skin). Screening for antibacterial activity toward *S. aureus*, narrowed the field of isolates down to four LAB strains with high antibacterial activity. The activity varied according to the specific LAB strain and the origin of the strain. The results suggested different modes of action, including co-aggregation, expression of bacteriocins and production of specific organic acids. However, the ability to acidify the surroundings appeared as the main effect behind inhibition of *S. aureus.* Broth microdilution assays showed a significant reduction of *S. aureus* growth when using down to 10% cell free supernatant (CFS). Our results underline the use of specific living LAB or their CFS as potential future treatment strategies to reduce *S. aureus* colonization of AD skin.

## Introduction

Atopic dermatitis (AD) is characterized by chronic cutaneous inflammation, epidermal barrier dysfunction and increased susceptibility to skin infections (Bieber, 2008; Brown, 2016). Colonization with *S. aureus* is found on the skin of ~70% of AD patients and is directly correlated with the severity of the disease (Totté et al., 2016; Geoghegan et al., 2018). Whether *S. aureus* is the primary trigger of inflammation or simply colonizes the skin due to inflammation is unknown, but reduction of *S. aureus* on the skin has been found to alleviate symptoms of the disease (Gong et al., 2006; Myles et al., 2018; Nakatsuji et al., 2021). A high proportion of *S. aureus* colonizing skin of AD patients are found to be resistant to the preferred topical antibiotic treatment with fusidic acid (Edslev et al., 2017) and alternatives to the traditional antibiotic therapies are therefore of interest.

Lactic acid bacteria (LAB) are a ubiquitous group of bacteria including lactobacilli, lactococci, enterococci, streptococci, leuconostoc, and pediococci. LAB are found in soil, water and plants but also on mucosal surfaces of humans and animals in the gastrointestinal and urogenital tracts, as well as on the human skin (Grice et al., 2009; Chu et al., 2017). LAB are generally considered beneficial due to their immunomodulating effect, their ability to alter the microbiota composition in some habitats and their positive effect on the gastro-intestinal health (Hill et al., 2014). LAB release bioactive molecules such as organic acids and antimicrobial peptides (AMP) that inhibit pathogen growth and interfere with the quorum sensing system of pathogens (Arief et al., 2015). Some LAB co-aggregate with pathogens facilitating removal of the pathogen from the skin via peristaltic elimination and thereby prevention of pathogen skin interaction (Lukic et al., 2017; Siedler et al., 2020). Another antimicrobial mechanism indicated by co-aggregation is competitive displacement of the pathogen with some LAB strains showing high affinity binding to epithelial cell receptors (Lukic et al., 2017; Spacova et al., 2020). Several studies demonstrate antimicrobial effects of LAB on *S. aureus* (Blanchet-Réthoré et al., 2017; Jayashree et al., 2018; Spacova et al., 2020; Delanghe et al., 2021; Musa et al., 2021) and recent findings indicate, that aggregation of lactobacilli to *S. aureus* is important for the ability of lactobacilli to prevent or reduce *S. aureus* adhesion to epithelial surfaces (Younes et al., 2016). Previous study has shown that topical application of LAB in a cream formulation can have beneficial effect on AD symptoms and *S. aureus* colonization on skin (Blanchet-Réthoré et al., 2017; Butler et al., 2020). Most studies target standard laboratory *S. aureus* without specifying the clonal type of *S. aureus*. However, findings indicate that a specific clonal lineage of *S. aureus*, clonal complex (CC) type 1, are more prevalent in AD patients, and are also associated with filaggrin gene (FLG) mutations (Irvine et al., 2011; Clausen et al., 2017). These findings suggest that the CC-1-type of *S. aureus* is important in colonization of the AD skin, and therefore a relevant target when studying the microbial interactions on the skin.

The aim of this study was to isolate novel LAB strains from several different habitats, establish a strain collection with substantial variation in lactobacilli species and screen for beneficial antibacterial properties toward AD prevalent *S. aureus* CC-type 1.

## Materials and Methods

**FIGURE 1:**
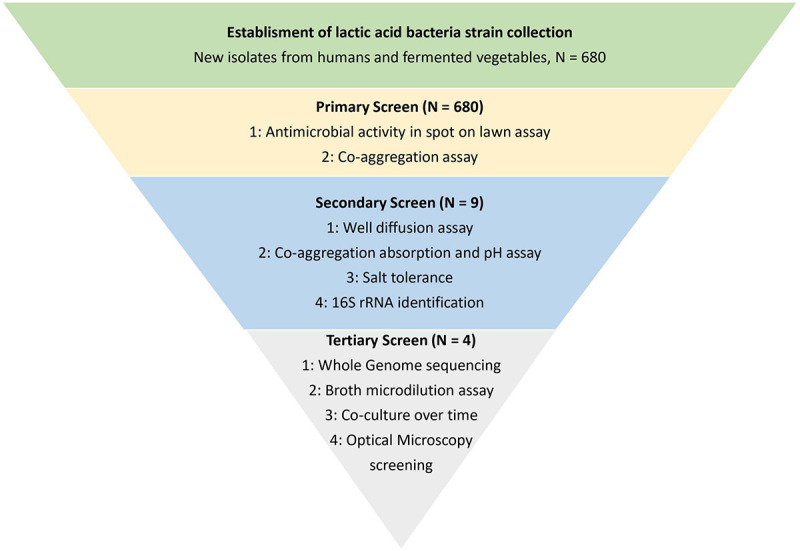
Schematic overview of the study. The study was divided into three screening parts for identification and characterization of lactic acid bacteria with antimicrobial activity toward *Staphylococcus aureus* CC-type 1. The screening was performed to narrow down the library of 680 lactic acid bacteria strains to four strains with the highest observed antibacterial activity.

The study was divided into three screening parts, enabling the discovery of LAB with antibacterial activity toward *S. aureus* from a library collection of 680 LAB. A flow chart of the screening process is given in Figure 1.

### Bacterial Strains and Growth Conditions

A library of 680 LAB was established for screening in this study. LAB were isolated from different natural sources, including fermented vegetables and swab samples from vaginal, stool and skin of Danish healthy volunteers (Table 1), in compliance with the national laws on access to genetic resources. All LAB strains in the library were collected in compliance with the Bioconvention and the Nagoya protocol (The Convention on Biological Diversity, 2011). The LAB were isolated on de Man, Rogosa and Sharp (MRS) agar plates (69964, Millipore) incubated at 37°C under anaerobic conditions for 24 h. Colonies were randomly picked from the agar plate and purified prior to long term storage in MRS broth (69966, Millipore) with 20% glycerol (G5516, Sigma Aldrich) at −80°C.

**TABLE 1:** Antimicrobial activity (as determined by spot on lawn assay and hydrogen peroxide production) against *Staphyloccous aureus* CC-type 1, co-aggregation with *S. aureus* CC-type 1, species level identification (by 16S rRNA gene sequencing) and salt tolerance of the 9 best performing LAB from the secondary screen.

**Isolation source**	**Isolate ID**	**16S rRNA gene sequencing**		**Co-aggregation assay (%)**		**Spot on lawn assay (mm radius)**			**Hydrogen peroxide**	**Salt tolerance (% NaCl)**
		**Strain**	Similarity to database^*^ (%)	**20°C, pH 7**	**20°C, pH 5**	***S. aureus* CC-type 1**	***S. hominis***	***S. epidermidis***		
Kimchi	LB10G	*Weissella viridescens*	100.00	26.07.4^a +^	41.432.5^a +^	3.30.8^a +^	3.7 ± 1.2^a+^	3.2 ± 1.3^a+^	+	1.5
Sauerkraut	LB113R	*Lactiplantibacillus plantarum*	99.13	32.317.9^a +^	40.531.4^a +^	0.20.3^b +^	0.0^b+^	0.0^b+^	*^–^*	3.0
Sauerkraut	LB116R	*Lacticaseibacillus paracasei*	99.46	27.110.4^*ab* +^	68.636.7^a +^	0.51.3^b +^	0.0^b+^	0.0^b+^	+	4.5
Sauerkraut	LB244R	*Lactiplantibacillus plantarum*	100.00	4.04.5^c +^	71.437.0^a−^	5.01.2^a +^	5.6 ± 1.8^a+^	4.0 ± 2.0^a+^	+	4.5
Ensilage	LB276R	*Enterococcus faecium*	98.82	23.610.5^a +^	56.140.0^a +^	0.80.7^b +^	0.7 ± 1.1^b+^	0.0^b+^	*^–^*	3.0
Fecal sample	LB312R	*Lactiplantibacillus plantarum*	100.00	11.513.0^*bc* +^	53.837.4^a +^	1.10.9^b +^	0.8 ± 0.8^bd+^	0.2 ± 0.4^b+^	*^–^*	4.5
Fecal sample	LB316R	*Lactiplantibacillus plantarum*	99.57	0.00.0^c +^	67.136.7^a−^	0.30.5^b +^	0.17 ± 0.4^b+^	0.0^b+^	*^–^*	4.5
Fermented kale	LB349R	*Leuconostoc mesenteroides*	100.00	7.15.6^c +^	56.533.9^a +^	1.21.1^*bc* +^	0.9 ± 1.5^bc+^	0.4 ± 0.2^b+^	*^–^*	4.5
Fermented beetroot	LB356R	*Lactiplantibacillus plantarum*	99.49	1.53.65^c +^	80.316.24^a−^	2.61.8^*acd* +^	3.5 ± 1.6^a+^	2.6 ± 1.7^ac+^	*^–^*	4.5

*EzBioCloud 16S rRNA database (Yoon et al., 2017). The percentage of co-aggregation was measured at pH 7 in PBS buffer and pH 5 in MES buffer. The radius of the inhibition zone in the spot assay was measured in mm. The values are means of triplicates ± standard deviation of the means. Lowercase letters represent one way ANOVA test with post hoc Tukey test. Equivalent lowercase letters marked abc, by column, mean no significant differences between isolates at each condition (p > 0.05). Equivalent lowercase letters marked + −, per row, mean no significant differences between each condition (p > 0.05).

*Staphylococcus epidermidis* HM-140 (BEI Resources), *Staphylococcus hominis* HM-119 (BEI Resources) and human origin isolate of *Staphylococcus aureus* clonal complex (CC) type 1 (University of Copenhagen) were used as target organisms in this study. The *Staphylococcus* strains were grown in Brain Heart Infusion (BHI) broth (53286, Millipore) at 37°C under aerobic conditions overnight.

### Visual Co-aggregation (Primary Screen)

Visual co-aggregation between LAB isolates and *S. aureus* CC-type 1 was performed in the primary screen of all 680 LAB strains as a rapid screening according to Cisar et al. (1979) and Ekmekci et al. (2009). LAB isolates were grown in MRS broth and target organism *S. aureus* CC-type 1 in BHI broth over night at 37°C under aerobic conditions. The cultures were harvested by centrifugation at 10,000 × g for 5 min, washed twice using sterile phosphate-buffered saline (PBS) buffer (P5493, Sigma Aldrich) and hereafter resuspended in PBS buffer. LAB suspension (200 μL) was mixed with 200 μL of the target organism *S. aureus* CC-type 1 in 48-well plates and incubated overnight at room temperature under constant shaking at ~500 rpm. Control samples containing 200 μl of each bacterial suspension were maintained at the same time. Co-aggregation was scored according to the degree of co-aggregation ranging from 0 (no-aggregation) to four (large co-aggregates covering most of the well) (Cisar et al., 1979).

### Spot on Lawn Assay (Primary Screen)

The spot on lawn assay was performed according to Zhang and Tamplin (2015) with some modifications. An overnight culture of *S. aureus* CC-type 1 was diluted to an OD of 1.0 (600 nm) and further diluted twofold. Two hundred microliter of the adjusted cell suspension was plated onto BHI agar plates (70138, Millipore) and the plates were left for drying for ~10 min. Overnight cultures of isolated LAB (20 μL) were spotted onto the dry *S. aureus* lawn. The inhibition halos around the spots were scored at four levels, 4, 3, 2 and 1, corresponding to radius (R) ≥ 4 mm, 2 mm ≤ R < 4 mm, 0.5 mm < R < 2 mm and 0 < R ≤ 0.5 mm, respectively (Zhang and Tamplin, 2015).

### Absorbance Detection of Co-aggregation (Secondary Screen)

Co-aggregation between LAB isolates and *S. aureus* CC-type 1 was determined by measuring optical density (OD) at 600 nm according to Handley et al. (1987). The percentage of co-aggregation is calculated by following equation.


$$Co-aggregation\left(\%\right)=\frac{\left(\left(O\,D_{t\,a\,r\,g\,e\,t}+O\,D_{L\,A\,B}\right)-2\,x\,O\,D_{m\,i\,x}\right)}{\left(O\,D_{t\,a\,r\,g\,e\,t}+O\,D_{L\,A\,B}\right)}$$

OD_*target*_, OD_*LAB*_, and OD_*mix*_ represent the OD measure at 600 nm of individual pathogen, LAB and their mixture after incubation for 24 h. Lowercase letters represent significant differences between the LAB strains (*p* < 0.05) (Handley et al., 1987).

### pH Effect on Co-aggregation (Secondary Screen)

According to Ekmekci et al. (2009) the pH of the solution can affect the co-aggregation ability. Hence, 2-(N-Morpholino) ethanesulfonic acid (MES) buffer (69892, Sigma-Aldrich) with a pH adjusted according to the pH of the skin (pH 5) was used instead of PBS for cell suspension and otherwise carried out as in section “Absorbance Detection of Co-aggregation (Secondary Screen).”

### Well Diffusion Assay (Secondary Screen)

A well diffusion assay was performed with overnight cultures of LAB and the cell free supernatant (CFS) of LAB. The assay was performed according to Oldak et al. (2017) with some modifications. Overnight cultures of LAB were centrifuged (8,000 × g, 20 min, 4°C) and the supernatant filter sterilized (Filtropur S 0.2 μm membrane, Sarstedt) to remove excess cells. The overnight culture and CFS was filled in 7 mm wells in Mueller-Hinton agar plates inoculated with *S. aureus* CC-type 1, *S. epidermidis* or *S. hominis* and the radius of the inhibition zone measured (in mm) in triplicate of three independent experiments.

### Hydrogen Peroxide Production (Secondary Screen)

Test for production of hydrogen peroxide was performed according to Marshall (1979). Overnight culture of isolated LAB was spotted (20 μL) onto MRS agar plates with 0.25 mg/ml 3,3′, 5,5′-tetramethylbenzidine (TMB) (Sigma-Aldrich) and 0.01 mg/ml of horseradish peroxidase (HRP) (Sigma-Aldrich). The plates were incubated at 37°C under anaerobic conditions for 24 h and hereafter exposed to oxygen in 4 h before visually determining hydrogen peroxide production due to blue pigmentation in the spots (Marshall, 1979).

### Salt Tolerance of LAB (Secondary Screen)

To test the survival ability of LAB on skin, the salt tolerance of the LAB hit strains at NaCl concentrations of 1.5; 3; 4.5; and 6% in MRS broth were used. Overnight culture of LAB hit strains were inoculated into the salt broth concentrations and incubated over night at 37°C aerobically. Growth of LAB in the MRS + NaCl was determined visually (clear well when no growth).

### Identification by 16S rRNA Gene Sequencing (Secondary Screen)

The isolates (9 in total) with most promising antimicrobial activity against the targeted *Staphylococcus* strains (see section “Bacterial Strains and Growth Conditions”) were identified by near full-length 16S rRNA gene sequencing (V1–V9 regions), sequencing services were provided by GENEWIZ Germany GmbH. Similarity-based search against EzBioCloud 16S rRNA gene sequence database was used for taxonomic identification (Yoon et al., 2017).

### Analysis of Whole Genome Sequences (Tertiary Screen)

Four of the LAB hit strains were whole genome sequenced by Baseclear (Leiden, Netherlands) and annotated using Rapid Annotation Subsystem Technology (RAST) server^1^ to reveal virulence or antibiotic resistance encoding genes. ResFinder^2^ was used subsequently to analyze the four genomes for resistance genes (Bortolaia et al., 2020). The analysis is set to search for acquired antimicrobial resistance genes. The annotation program Bacteriocin Genome mining tool, BAGEL4^3^ was used to reveal potential bacteriocin encoding genes (Van Heel et al., 2018).

### Co-culture Assay—Inhibition Over Time (Tertiary Screening)

The antibacterial effect of LAB toward *S. aureus* CC-type 1 was determined over time according to Coman et al. (2014) and Tetili et al. (2017) with modifications. A suspension of 2 mL MRS broth mixed 1:4 with overnight LAB culture (~10^6^ CFU/ml) was mixed with 2 mL BHI broth with 1% overnight culture of *S. aureus* CC-type 1 (~10^6^ CFU/ml). The antibacterial effect of LAB was revealed by CFU counting of viable *S. aureus* on selective mannitol salt phenol red agar (MSA) (Millipore 63567) according to Jett et al. (1997). The CFU/ml of *S. aureus* CC-type 1 was determined after 0, 2, 4, 8, 12, and 24 h of incubation.

### Turbidimetric Assay of S. aureus and LAB Cell Free Supernatant (Tertiary Screening)

A broth microdilution assay with various fractions of LAB CFS was used to determine the minimum percentage of CFS able to inhibit growth of *S. aureus*. The growth inhibition was measured by phase contrast microscopy and image analysis using an oCelloscope (BioSense Solution, Denmark). The inhibitory effect of LAB CFS on *S. aureus* CC-type 1 was measured over time as described previously with slight modifications (Fredborg et al., 2013). An overnight culture of *S. aureus* was diluted to a concentration of ~10^4^ CFU/ml. An overnight culture of LAB (10^9^ CFU/ml) was filtered through a 0.2 μm filter to remove all cells. The CFS was diluted into 75, 50, 25, and 10% of the original content using MRS broth. A 100 μL aliquot of diluted *S. aureus* cell suspension was mixed with 100 μL undiluted or diluted CFS in 96 well plates. The plate was sealed with oxygen permeable film (Sigma-Aldrich) and incubated in the oCelloScope instrument (BioSense Solution, Denmark) at 37°C for 18 h. The *S. aureus* growth was measured every 20 min as segmentation and extraction of surface area (SESA).

### Statistical Analysis

Statistical comparisons were performed by one-way ANOVA test (*p* < 0.05 was considered as statistically significant). Tukey’s test was used to perform multiple comparisons between all means. All statistical studies were conducted in the statistical software R (version 4.0.0. R Core Team, 2020, Vienna, Austria).^4^

## Results

### Primary Screen

**FIGURE 2:**
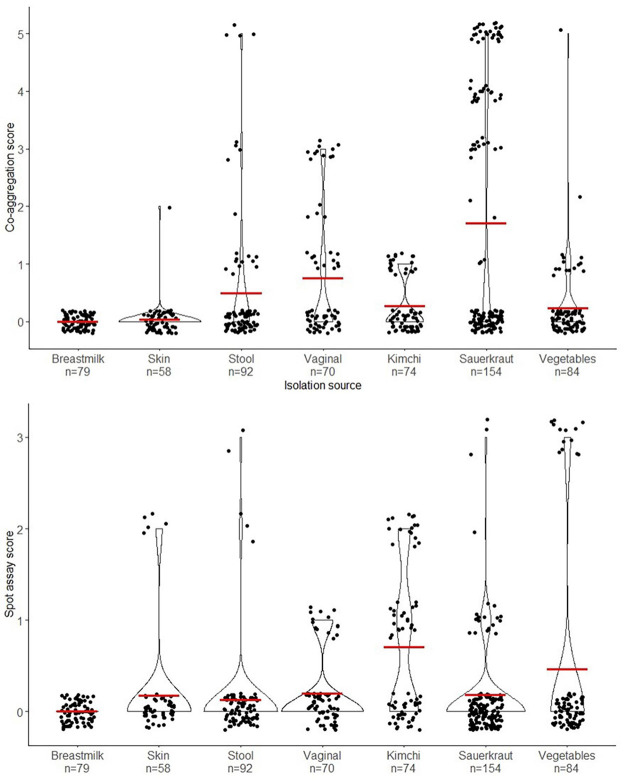
Violin plot illustrating the distribution of all the isolates from the primary screen and their isolation source. The upper graph shows the distribution according to co-aggregation score (0–5) and the lower graph shows the distribution of the isolates according to the spot on lawn assay score (0–5). The red crossbar shows the mean of the assay scores for each isolation source.

A total of 680 presumptive LAB isolates were cultivated on MRS agar from fermented vegetables (sauerkraut, Kimchi, and fermented vegetables) and from swab samples from healthy male and female Danish volunteers (vaginal, stool, and skin samples) and investigated for their antibacterial effect against *S. aureus* CC-type 1. As seen from Figure 2 isolates varied widely in this as determined by spot on lawn and co-aggregation assay. The outcome depended on source of origin and the specific strain. Especially LAB isolated from sauerkraut showed a high co-aggregation score while isolates from kimchi showed a high antimicrobial activity (Figure 2). Five strains with the highest observed co-aggregation abilities and four with the highest antibacterial activity on the spot assay were selected for further identification and characterization (Figure 1).

### Secondary Screen

#### Identification by 16S rRNA Sequencing

Five of the nine isolates selected for further characterization were identified as *Lactiplantibacillus plantarum*, one as *Lacticaseibacillus paracasei* and the three remaining isolates were identified as *Weissella viridescens*, *Enterococcus faecium*, and *Leuconostoc mesenteroides*, respectively (Table 1).

#### Spectrophotometric Co-aggregation Assay

The LAB isolates that showed high co-aggregation score in the primary screen were studied thoroughly by measuring the percentage of co-aggregation (Table 1). *W. viridescens* LB10G, *Lp. plantarum*, LB113R and LB116R, and *Enterococcus faecium* LB276R showed the highest percentage of co-aggregation (*p* < 0.05) compared to the other hit strains at pH 7 (Table 1). When the pH was lowered to pH 5 to mimic the pH of the skin, the co-aggregation increased for all hit strains and considerably for *Lp. plantarum* LB244R, LB316R, and LB356R (*p* < 0.05).

#### Antibacterial Activity

The antibacterial effect of the nine most promising LAB was tested against three different staphylococci species, namely *S. aureus* and coagulase negative *S. epidermidis* and *S. hominis* (Table 1). *W. viridescens* LB10G, *Lp. plantarum* LB244R and LB356R showed the strongest inhibition toward all three species of staphylococci (Table 1). On the contrary, *Lp. plantarum* LB113R and LB116R which had the highest co-aggregation ratio exerted limited antibacterial activity toward the target organisms. No significant difference in antibacterial activity of LAB against the three Staphylococci species were found.

#### Hydrogen Peroxide Production

Of the nine hit strains, *W. viridescens* LB10G, *Lc. paracasei* LB116R, and *Lp. plantarum* LB244R showed hydrogen peroxide production (Table 1).

#### Stress Tolerance Toward NaCl

To simulate survival under the relatively high NaCl concentrations bacteria might encounter on the skin, the nine hit strains were tested for their salt tolerance. *Lp. plantarum* LB244R, LB312R, LB316R, and LB356R showed the highest tolerance (4.5% added NaCl) while *W. viridescens* LB10G showed the lowest tolerance (1.5% added NaCl) (Table 1).

### Tertiary Screen

From the secondary screen, four of the LAB strains, namely *W. viridescens* LB10G, *Lp. plantarum* LB113R, and *Lp. plantarum* LB244R and LB356R, were chosen for further characterization. The strains were selected due to their high co-aggregation abilities or due to their high antibacterial activity in the well diffusion assay.

#### Basic Genome Sequence Analysis of LAB Strains

Several genes involved in bacteriocin production were identified in the *Lp. plantarum* LB244R and LB356R genome sequences (Supplementary Table 1). These genes are located in the well characterized bacteriocin plantaricin (pln) loci consisting of operons with plantaricin encoding genes and genes involved in the three-component regulatory networks (Diep et al., 2009). The loci found in *Lp. plantarum* LB244R contains the operons plnGHSTUVW, plnABCD, and plnEFI, indicating a quorum sensing regulation of the plantaricin EF complex and the cognate immunity protein by the three-component regulatory network, consisting of the inducing factor plantaricin A, the histidine kinase plnB and the two regulators plnC and plnD (Diep et al., 1995, 2009; Nes et al., 1996; Goel et al., 2020). The plnEF complex was found in *Lp. plantarum* LB356R as well, along with plnJK and plnN. No known bacteriocin encoding genes were found in *W. viridescens* LB10G and *Lp. plantarum* LB113R.

Additionally, genes involved in the production of antibacterial metabolites were found in the genome of all four strains. Hydroxyisocaproate dehydrogenase responsible for the conversion of 2-hydroxyisocaproic acid (HICA) was found in *Lp. plantarum* LB244R and LB356R. HICA has previously been identified as antibacterial toward *S. aureus* (Park et al., 2017).

**FIGURE 3:**
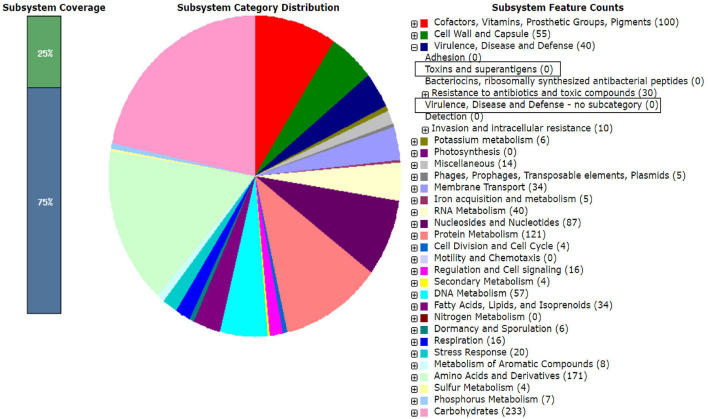
RAST analysis on the genome sequence of the *Lactiplantibacillus plantarum* subsp. *plantarum* LB244R. The marked boxes show that no genes encoding toxins or virulence were found in the genome.

The RAST analysis of the four LAB genomes did not reveal any virulence or toxin encoding genes (Figure 3). Genes encoding tetracycline and beta-lactam resistance were found in the RAST analysis in conserved domains of LB244R and LB356R, however, these genes were not classified as being functional and additional analysis with the server ResFinder did not reveal any resistance genes in the four strains (Bortolaia et al., 2020). Minimum inhibitory concentration (MIC) assays with tetracycline and ampicillin did not reveal resistance, according to the cut-off values determined by the European Food Safety Authority (EFSA, 2012) (data not shown). Beta-lactamase resistance gene was found in LB113R, however, MIC assays showed susceptibility toward ampicillin. No resistance genes were found in *W. viridescens* LB10G.

#### The Four Hit Strains Inhibit S. aureus CC-Type 1 in Co-culture

**FIGURE 4:**
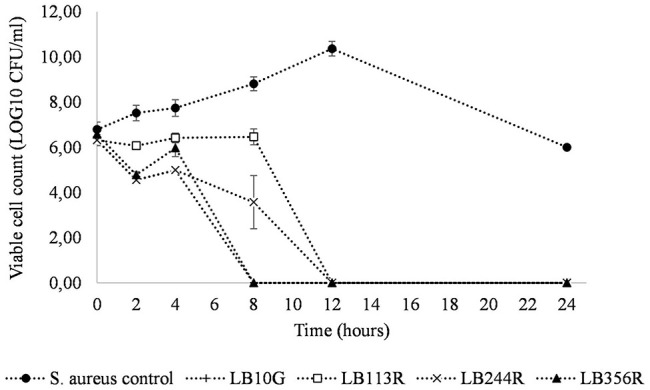
Co-culture of *Staphylococcus aureus* CC-type 1 and the four hit strains *Lactiplantibacillus plantarum* subsp. *plantarum* LB113R, LB244R and LB356R and *Weissella viridescens* LB10G over time. The growth of *S. aureus* is detected on *S. aureus* selective MSA agar plates with a detection limit of 100 CFU/g. The values are the mean ± standard deviations of triplicates repeated in two individual experiments.

The inhibitory effect of the four LAB strains on *S. aureus* CC-type 1 was determined during 24 h in a co-culture assay (Figure 4). After 4 h of incubation, the viable cell count of *S. aureus* CC-type 1 was significantly reduced in the co-cultures for all four strains (2.5–3.0 log CFU/ml, *p* < 0.001). After 8 hs of incubation no viable cells were detected in the co-culture with LB356R and after 12 h of incubation, no viable *S. aureus* cells were detected for all four co-cultures (detection limit 100 CFU/g).

#### Cell Free Supernatant of LAB Inhibits S. aureus CC-Type 1

**FIGURE 5:**
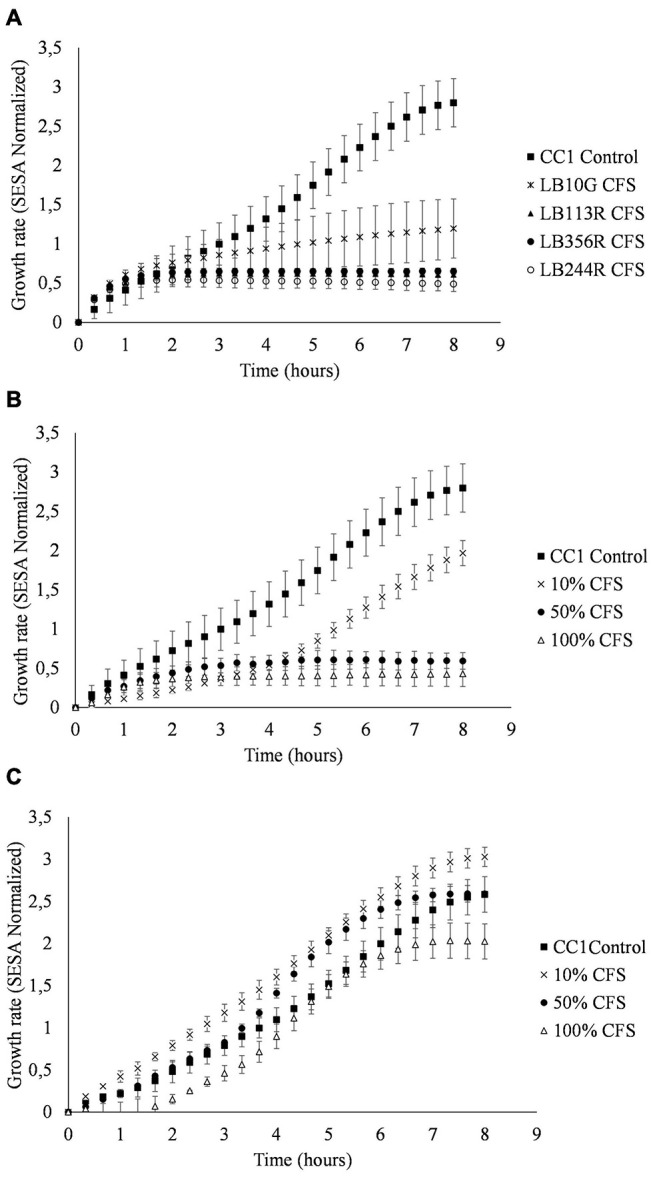
Culture assays measuring the growth of *S. aureus* CC-type 1 when exposed to cell-free supernatants from *Lactiplantibacillus plantarum* subsp. *plantarum* LB224R. Growth over time assessed by the oCelloscope using the normalized segmentation and extraction of surface area (SESA) algorithm. **(A)** *S. aureus* CC-type 1 growth inhibition by CFS from the LAB strains selected in the tertiary screen **(B)** Broth microdilution determination of *S. aureus* CC-type 1 growth inhibition by percentages of added LB244R CFS. **(C)** Determination of pH effect on antibacterial activity of CFS. *S. aureus* CC-type 1 is cultured with pH neutralized LB244R CFS (pH 6). The values are the mean ± standard deviations of triplicates repeated in two individual experiments.

CFS from all four LAB strains selected in the tertiary screen inhibited growth of *S. aureus* CC-type 1 (Figure 5A). *Lp. plantarum* LB113R as well as *Lp. plantarum* LB244R and LB356R showed complete inhibition after 2 h. *W. viridescens* LB10G did not inhibit *S. aureus* CC-type 1 to the same extent, but growth of *S. aureus* CC-type 1 was still significantly reduced from 2.79 SESA to 1.19 SESA (*p* < 0.001) after 8 h of incubation. Broth microdilution of *Lp. plantarum* LB244R and LB356R CFS showed reduction in growth using as low as 10% CFS (Figure 5B). The lag phase of the CC-type 1 was prolonged when adding 10 and 25% of *Lp. plantarum* LB244R and LB356R CFS to the culture, however, after 12 h of incubation, the growth was equal to the control sample. Complete inhibition of growth was detected using 100 and 50% of *Lp. plantarum* LB244R and LB356R CFS.

To adjust for pH effect of the CFS on the viability of *S. aureus* CC-type 1, the pH of the CFS was neutralized to around pH 6. When the pH of the CFS was adjusted, the inhibitory effect of the CFS was lost for all four LAB strains when using 10 and 50% CFS. A prolonged lag phase and slightly reduced growth from 2.58 to 2.02 SESA (*p* = 0.365) was detected when using 100% pH neutralized CFS (Figure 5C).

## Discussion

The present study selectively identified novel isolated LAB strains potentially suitable for reduction of *S. aureus* colonization of AD skin. The antibacterial effect was tested against *S. aureus* CC-type 1, which is the most prevalent CC-type in AD (Clausen et al., 2017).

The isolates showing the highest antibacterial effect toward *S. aureus* were primarily LAB isolated from fermented vegetables, with LAB isolated from human swab samples being less inhibitory on average (Figure 2). This indicates that the microenvironment of the bacteria, may lead to a differentiation of the competitive mechanisms between species according to their natural microenvironment (Oldak et al., 2017). LAB isolated from sauerkraut (salt concentration of 1.5–3 %) revealed a rather high salt tolerance of 4.5%, suggesting that the LAB isolates would be capable of prolonged survival on the skin environment with high salt concentrations.

The antibacterial effect of LAB was tested toward *S. hominis* and *S. epidermidis*, two skin commensals, to study whether the effect specifically targets *S. aureus* or a variety of skin bacteria. The results showed antibacterial effect toward all three staphylococci, with no significant difference (*p* < 0.05) in inhibition among them (Table 1). The antibacterial effect of LAB toward *S. aureus* was very specific to the individual LAB isolate. The findings showed that *Lp. plantarum* LB244R and LB356R had the highest antibacterial activity (Table 1) while the isolates LB113R and LB116R showed good co-aggregation abilities (Table 1), but very little antibacterial effect in the spot tests (Table 1). Overnight culture of all four strains had an acidic pH of ~3.8 but only *Lp. plantarum* LB244R and LB356R showed antibacterial effect in the spot test (Table 1). These differences in activity suggest that the inhibitory effect is not only due to lowered environmental pH. Analysis of the genome sequences of LB244R and LB356R revealed genes encoding the bacteriocin plantaricin EF and JK complex. Further analysis would be to test whether these are expressed by the LAB cultures and if the expression is upregulated upon exposure to *S. aureus*. Selegård et al. (2019) have showed that plantaricins EF and JK significantly lyse *S. epidermidis* and following studies by Musa et al. (2021) showed how the two-peptide Plantaricin NC8 αβ eliminated *S. aureus* and counteracting its inflammatory and cytotoxic effect (Selegård et al., 2019; Musa et al., 2021). No bacteriocin encoding genes were found in the genome of LB113R, supporting the spot assay results revealing no antibacterial effect of LB113R. No effect was observed for the CFS or heat-treated LAB cells on the spot assays (data not shown), suggesting that the growth conditions or the direct interaction between the LAB and *S. aureus* on the spot agar plate affect the production of antibacterial compounds. The liquid co-culture of LAB CFS and *S. aureus* CC-type 1 showed a high antibacterial effect of the LAB CFS in contrast to the spot assay on solid agar. The antibacterial effect of the LAB CFS was lost when pH was neutralized indicating most of the effect being due to acidic pH and organic acids produced by LAB.

The results of the optical screening with LAB CFS indicate the importance of acidic pH to maintain antibacterial effect of the bioactive compounds found in the CFS. Neutralized pH can affect the produced bacteriocins and the effect of these. Studies by Barbosa et al. (2016) showed that plantaricins lost their activity at a pH higher than pH 6, indicating that changing the pH alters the effect of the bacteriocins in the CFS (Barbosa et al., 2016). Additionally, organic acids can, in their undissociated form, penetrate the cytoplasmic membrane, causing intracellular acidification and a following collapse of the transmembrane proton motive force (Arena et al., 2016).

Production of organic acids by LAB may have a positive effect on the AD skin (Lee et al., 2016). The pH of AD skin infected with *S. aureus* is found to be higher than the pH of normal skin, which increases the vulnerability of the skin barrier and might promote *S. aureus* colonization (Ali and Yosipovitch, 2013; Panther and Jacob, 2015; Clausen et al., 2019). According to Miajlovic et al. (2010), lowered pH led to the reduction of protein expression of surface proteins promoting colonization of host tissue by *S. aureus*. Hence, the acid produced by LAB might also affect the pH of the skin and hereby decrease the colonization of *S. aureus*.

Specific clonal lineages of *S. aureus* are detected in AD patients, with *S. aureus* CC-type 1 as the most prevalent among AD patients with filaggrin mutations (Clausen et al., 2019; Iwamoto et al., 2019). Recent studies have shown that in comparison to standard laboratory *S. aureus* strains, clinical *S. aureus* strains from AD skin promote an AD-specific immune environment by altering the T cell response resulting in a Th2/Th22 skewed immune response (Iwamoto et al., 2017, 2019) suggested that the *S. aureus* derived cell wall proteins and secreted virulence factors represent a future therapeutic target (Iwamoto et al., 2019). Future studies could be to investigate the effect of LAB on the expression of these virulence factors and whether the LAB through microbial interactions could shift the gene expression of *S. aureus* toward a commensal state.

While RAST-based analysis of the whole-genome sequenced isolates showed that isolates LB244R and LB335R carried genes encoding resistance against tetracycline and beta-lactams in conserved regions of their genome and isolate LB113R carried genes encoding resistance against beta-lactams. However, more detailed inspection of the genomes using ResFinder could not confirm this. Further, when tested against tetracycline and ampicillin, these isolates were all susceptible at concentrations well below the cut-off values recommended by the European Food Safety Authority (EFSA, 2012). Horizontal transfer of genes encoding antibiotic resistance from these strains does therefore not seem to be risk. The ability to decarboxylate amino acids is a common trait among LAB and might result in the formation of biogenic amines (Barbieri et al., 2019). The isolates obtained in the present study was not tested for their ability to produce biogenic amines. However, as the isolates are intended for use as skin targeting probiotics, the ability to form biogenic amines does not seem like a major risk either.

The strength of this study is the large library of LAB with broad diversity of origin and how this affects their antimicrobial activity. Subsequently, the screening was targeted specifically toward *S. aureus* CC-type 1, which is dominant on the skin of AD patients. However, the results presented remain preliminary as the isolates have not yet been tested *in vivo*. Furthermore, future studies are required to illuminate in more detail how the LAB will affect and interact with the AD skin cells upon colonization of *S. aureus* CC-type 1.

## Conclusion

In summary, four LAB candidates were found to inhibit growth of *S. aureus* CC-type 1. The results indicate that the antibacterial activity is caused primarily by acidic pH and might be due to a combination of different modes of action including co-aggregation, bacteriocin production and other antibacterial metabolites. These findings suggest that specific LAB strains hold potential as live biotherapeutic preventing *S. aureus* colonization of AD skin.

## Data Availability Statement

The data presented in the study are deposited in the GenBank repository, accession numbers JAIFOK000000000 (LB10G), JAIFOL000000000 (LB113R), MZ855496 (LB116R), JAIFOR000000000 (LB244R), MZ855497 (LB276R), MZ855498 (LB312R), MZ855499 (LB316R), MZ855500 (LB349R), and JAIFOM000000000 (LB356R).

## Author Contributions

SK, CV, TA, M-LC, and IC formulated and designed the study. IC performed the experimental work, described in this manuscript, processed, analyzed the data, and prepared the manuscript. DN carefully edited the first draft of the manuscript. All authors interpreted the experimental results along with the preparation of the manuscript draft, and gave their approval to the final version of the manuscript. The manuscript was written through contributions of all authors.

## Conflict of Interest

SK and CV have financial relationships as CEO and COO of Lactobio A/S. IC is an employee of Lactobio. The remaining authors declare that the research was conducted in the absence of any commercial or financial relationships that could be construed as a potential conflict of interest. The authors declare that this study received funding from Lactobio A/S. The funder had the following involvement in the study: study design, data collection and analysis, decision to publish and preparation of the manuscript.

## Publisher’s Note

All claims expressed in this article are solely those of the authors and do not necessarily represent those of their affiliated organizations, or those of the publisher, the editors and the reviewers. Any product that may be evaluated in this article, or claim that may be made by its manufacturer, is not guaranteed or endorsed by the publisher.

## Footnotes

1
http://rast.nmpdr.org/

2
https://cge.cbs.dtu.dk/services/ResFinder/

3
http://bagel4.molgenrug.nl/index.php

4
https://www.R-project.org/
