# Supplementary material for: Targeted Screening of Lactic Acid Bacteria With Antibacterial Activity Toward Staphylococcus aureus Clonal Complex Type 1 Associated With Atopic Dermatitis
Source: Front Microbiol. 2021 Sep 17;12:733847. doi: 10.3389/fmicb.2021.733847 (PMC8486014; doi:10.3389/fmicb.2021.733847)
Supplement: Supplementary file 1 [file Table_1.pdf]

Table S1 Plantaricin genes found in the genome of *Lactiplantibacillus plantarum* subsp. *plantarum* LB244R. BAGEL4 bacteriocin web server was used for genome annotation (Van Heel et al., 2018)

| **Name** | **Function** | **E-value Match (%) (BAGEL4)** | **Amino acid sequence** |
|---|---|---|---|
| PlnG / LanT | Bacteriocin ABC transporter, ATP-binding and permease protein PlnG | E-value 0.0<br>Match 100.0% | MHWRNYVAQVDEMDCGVAALAMILKNYGSTTSLAYLRNIAKTSLEGTTALGLVKTAEKLGF<br>ETKAIQADMSLFEVQDLPLPFIVHVTKNGDLQHFYVVVKTSKTHVVIADPDPTVAVISMSKERF<br>ESEWSGVALFFAPKSEYKPVKQDKGSLWGFIPSLLKQRRLVINIVLAAVLITIISICGSYFLQAVI<br>DTYIPNNMHSTLAVVAIGLIVFYTFQAIFTYAQNFLLAVLGQRLSIEIILGYIRHVFELPMSFFAT<br>RRTGEIVSRFTDASKIIDALASTIVSLFLDVSIVIIMGAILVIQNMTLFWITLLSLPIYAVVILAFNK<br>SFERLNQKEMESNAILSSAIIEDLHGIETVKALNGETERYQKIDSEFVDYLRKSLAYLKADTLQQ<br>ALKLFIQLVLEVVVLWVGANLVIHNQLSVGELMTYNALLAYFVNPLQNIINLQTKLQSAKVAN<br>NRLNEVYLVASEFEASRPIHNESQLNGDIKLQGVSYRYGYGENVLDDVNLTIRQHDKVAIVGM<br>SGSGKSTLVKLLIDFYQPNSGDVILNGFNVKNIDKHTLRTHINYIPQEPYIFSATIEENLRLGNRS<br>GITEKDIAKACQLALIDTDINKMAMQYQTKLDENGNTLSGGQRQRLTIARALLSPAQVLIFDES<br>TSGLDAITEKQLIDNLVAMTDKTIIFIAHRLSIAKRTNHIIVLHDGRVAEEGTHAALLNEHGYYY<br>DLINS |
| PlnE | Bacteriocin Plantaricin E | E-value 3.77e-38<br>Match 100.0% | MLQFEKLQYSRLPQKKLAKISGGFNRGGYNFGKSVRHVVDAIGSVAGIRGILKSIR |
| PlnF | Bacteriocin Plantaricin F | E-value 3.59e-36<br>Match 100.0% | MKKFLVLRDRELNAISGGVFHAYSARGVRNNYKSAVGPADWVISAVRGFIHG |
| PlnI | Immunity protein PlnI, membrane-bound protease CAAX family | E-value 6.90e-180<br>Match 99.6% | MGGILTMPLIKVIITAIYYLIVLFLMNPLTDIMGIKDGPFQFILTESIILIAIIILNRRYVKQPIHWLP<br>VNIMSILKKNSLPLSLAIIFLLIFFRNHMYQFLISLLLSLIVAITEEYAFRGMIFRTLLALNLKKFAT<br>LQATIASMMTASLIFAAMHLVNLLSQPVWSVLCQVLYVIGLGILLAAIYLKTGSLLAAISVHWL<br>IDFSSFYSQGIAPTQSPINGPMEALLKGLFLNILFIGIATFILSSKHWKLLSILNIEDKIDE |
| PlnD | Response regulator PlnD, repressor | E-value 2.1e-173<br>Match 99.2% | LFPIYLYEDNAEQRDNYCKTVNNTIMINEFAMELRVATDDQKIILADLNQQQDGLFFLDMEIGE<br>DKQTGLELASRIRATIPLAKIVFITTHDELSFVTLERRIAPLDYILKDQSADLITQRIIKDINVVQN<br>ELKKTNSQRKDVFNYKLGTRYFSLALDDVILLSTSKLRPGSVQLHAINKVAEFPGNLNALEEK<br>YPQFFRCDKSSLVNLNHLRSFDYKEKELLLDGEIRCKASFRKSRELNKLLRDN |

| | | | |
|---|---|---|---|
| PlnC | Response regulator PlnC, activator | E-value 4.17e-164 Match 100.0% | MIKEYDMQLVVATGDLQELMNNVMNSKEGLFFLDMEIGEQTQAGLNLADEIRQQLPCAQIVFITTHEELSFLTLERRIAPLDYILKEQGLDDIKQKIVKDIDATQTILKTETVQHKDILGYKIGTRFFSVPINDVIMLSTNKERPGSIRLTAKNKVADFPGNLNSFENKYSQFFRCDKSSLVNIDYVDSYDYQKKELTMIDNIKCSVSYRKSRELNKILKKK |
| PlnB | Bacteriocin production related histidine kinase | E-value 8.90e-18 Match 27.1% | LVEISIFDSVIQSFFIYLGIILVYNFIFSNTNIKRIIYSLILLLVLSLVGAILDDTTSLILVLRAIIKEKCQPKINYYHLNVFLMLISSQIVILALASYLSRGFLYIYLDADKISGLSEYGDLFIGIEIIVMYIIGFLVFNLVYKMVRRYTGSFDISDDERINRHLFIILLAFFGSIEMLLFISNFQGVTATIQLTLLLTFVLMLGLISWQTLETIRVYAWQKKIAAEKLQNKQLNDYLKSVEHQYLELRKFKHDYKNLIASLNTQDNISEIKDYLTDYTQSGEFRASLNDGSIASVQHLKNEILRGLVVQKFFYAKQCGVKLTIEIANTDFILSHGVTVAVRIIGNLLDNAIEQAQKMTDKIVTVAFNEIDNTAEIAISNPIDSDFNQHQIFETGYSTKGSNRGLGLTNVRDLVEQQKGFYMDIETKKNYVTMTLIVTEDK |
| PlnA | Induction pheromone, Plantaricin A | E-value 1.14e-29 Match 97.9% | MKIQIKSMKQLSNKEMQKIVGGKSSAYSLQMGATAIKQVKKLFKKWGW |
| PlnL | Putative bacteriocin Immunity | E-value 3.78e-21 Match 30.6% | MIEIDFKRRYYPLVLVLFVAGYMGAIFLNIYFDWLAMLTAGIISLVMLYGFSNLKLFFQPLPSPKWRIVMKYVLLLFVLEVTAIVVIAIINSHALLSFHNTTLTTNSTGRDYPLIERVWIAFTLLVSLVGEEVGMASISIPLIRLLSQTQLKKYAWPIINVLGCIVFACLHLPHYHFNWLYPLIVGITRYPITASWRSANTLRSGIYVHWISDAVLIIGTLI |
| PlnJ | Bacteriocin J | E-value 1.08e-11 Match 72.4% | MTVNKMIKDLDVVDAFAPISNNKLNGVVGGGAWKNFWSSLRKGFYDGEAGRAIRR |
| orf00043 | ComC; Lactococcin; Bacteriocin IIc | E-value 1.01e-13 Match 53.2% | MKNINNFQALQKNELSKVKGGSNNKFWTWAGYTYENWRISSRRAFNLRQRKNTMTHHVIKMKNINNFQALQKNELSKVKGGSNNKFWTWAGYTYENWRISSRRAFNLRQRKNTMTHH |
